# Humoral and T cell responses to SARS-CoV-2 reveal insights into immunity during the early pandemic period in Pakistan

**DOI:** 10.1186/s12879-023-08829-1

**Published:** 2023-12-01

**Authors:** Kiran Iqbal Masood, Shama Qaiser, Syed Hani Abidi, Erum Khan, Syed Faisal Mahmood, Areeba Hussain, Zara Ghous, Khekahsan Imtiaz, Natasha Ali, Muhammad Hasan, Haris Ali Memon, Maliha Yameen, Shiza Ali, Sadaf Baloch, Gulzar Lakhani, Paula M. Alves, Najeeha Talat Iqbal, Kumail Ahmed, Junaid Iqbal, Zulfiqar A. Bhutta, Rabia Hussain, Martin Rottenberg, J. Pedro Simas, Marc Veldhoen, Kulsoom Ghias, Zahra Hasan

**Affiliations:** 1 Department of Pathology and Laboratory Medicine, Aga Khan University, Stadium Road, P.O.Box 3500, Karachi, 74800 Pakistan; 2 Department of Biological and Biomedical Sciences, Aga Khan University, Karachi, Pakistan; 3 Department of Medicine, Aga Khan University, Karachi, Pakistan; 4 iBET - Instituto de Biologia Experimental E Tecnológica, Oeiras, Portugal; 5 Department of Pediatrics, Aga Khan University, Karachi, Pakistan; 6 Center of Excellence in Women and Child Health, Aga Khan University, Karachi, Pakistan; 7 Centre for Global Child Health, Hospital for Sick Children, Toronto, Canada; 8 Department of Microbiology and Tumor Cell Biology, Karolinska Institute, Stockholm, Sweden; 9 Católica Biomedical Research, Católica Medical School, Universidade Católica Portuguesa, Palma de Cima, 1649-023 Lisboa, Portugal; 10 Instituto de Medicina Molecular | João Lobo Antunes, Faculdade de Medicina, Universidade de Lisboa, Lisbon, Portugal

**Keywords:** Spike, Receptor binding domain, SARS-CoV-2, IgG, T cells, Interferon-gamma

## Abstract

**Background:**

Protection against SARS-CoV-2 is mediated by humoral and T cell responses. Pakistan faced relatively low morbidity and mortality from COVID-19 through the pandemic. To examine the role of prior immunity in the population, we studied IgG antibody response levels, virus neutralizing activity and T cell reactivity to Spike protein in a healthy control group (HG) as compared with COVID-19 cases and individuals from the pre-pandemic period (PP).

**Methods:**

HG and COVID-19 participants were recruited between October 2020 and May 2021. Pre-pandemic sera was collected before 2018. IgG antibodies against Spike and its Receptor Binding Domain (RBD) were determined by ELISA. Virus neutralization activity was determined using a PCR-based micro-neutralization assay. T cell – IFN-γ activation was assessed by ELISpot.

**Results:**

Overall, the magnitude of anti-Spike IgG antibody levels as well as seropositivity was greatest in COVID-19 cases (90%) as compared with HG (39.8%) and PP (12.2%). During the study period, Pakistan experienced three COVID-19 waves. We observed that IgG seropositivity to Spike in HG increased from 10.3 to 83.5% during the study, whilst seropositivity to RBD increased from 7.5 to 33.3%. IgG antibodies to Spike and RBD were correlated positively in all three study groups. Virus neutralizing activity was identified in sera of COVID-19, HG and PP. Spike reactive T cells were present in COVID-19, HG and PP groups. Individuals with reactive T cells included those with and without IgG antibodies to Spike.

**Conclusions:**

Antibody and T cell responses to Spike protein in individuals from the pre-pandemic period suggest prior immunity against SARS-CoV-2, most likely from cross-reactive responses. The rising seroprevalence observed in healthy individuals through the pandemic without known COVID-19 may be due to the activation of adaptive immunity from cross-reactive memory B and T cells. This may explain the more favourable COVID-19 outcomes observed in this population.

**Supplementary Information:**

The online version contains supplementary material available at 10.1186/s12879-023-08829-1.

## Background

Severe acute respiratory syndrome coronavirus 2 (SARS-CoV-2) causes a respiratory infection which is asymptomatic or minimal disease, in most individuals but can also cause moderate to severe disease leading to pneumonia [1]. The COVID-19 pandemic between 2020 and 2023 affected all countries of the world however, with variable morbidity and mortality between regions. The case fatality rate (CFR) from COVID-19 at the peak of the first wave of the pandemic in March 2020 ranged from 6.2% in Italy, 3.6% in Iran, to 0.79% in South Korea [2, 3]. Differences in disease severity and morbidity were also related to successive pandemic surges, later associated with SARS-CoV-2 variants and then, COVID-19 vaccinations. It is important to investigate the factors that impact such outcomes, especially the mechanisms of protection against COVID-19.

In South Asian countries such as Pakistan, relatively low COVID-19 morbidity was observed, with a CFR of about 2% even in the early phase of the pandemic [4]. In a population of 220 million, there have been 1.58 million positive cases with 30,646 COVID-19 related deaths (up to 25 March 2023) [5]. While most fatalities are associated with advanced age and underlying medical comorbidities [5, 6] both host and pathogen-related factors likely drive the immune protection against infection and severity in COVID-19.

SARS-CoV-2 has structural and non-structural proteins with varying functions. Membrane (M) proteins and Envelop (E) proteins are important in maintaining the structure, the Nucleocapsid (N) protein is primarily involved in replication while the Spike glycoprotein is involved in binding to the host receptor and entry into host cells [7]. Spike has two domains S1 and S2. S1 has the receptor binding domain (RBD) specific for ACE2 on human cells whilst, S2 provides fusion and entry properties [8]. During COVID-19, IgM antibodies to Spike are detectable within 5 to 7 days of the infection and IgG antibodies appear after 7 or more days, reaching a peak by day 14 [9, 10]. Strong correlation is observed between IgG antibodies and neutralizing activity against SARS-CoV-2 lasting up to 7 months [9].

In the early pandemic period of 2020, antibody seroprevalence studies showed positivity rates to vary between 0.66% (Hungary) to 21% (Iran) [11]. Seroprevalence to SARS-CoV-2 was seen to rise throughout the pandemic, with antibodies maintained for six months or more [12]. Raised seropositivity has been associated with high population densities, rapid transmission and infection rates [13]. A study of healthy blood donors in Karachi revealed a seropositivity of 53.4% to Spike protein and 16.7% to RBD by the end of 2020 [14].

SARS-CoV-2 infection induces T cell memory responses which result in long-lasting immunity [15]. Longitudinal analysis of B and T cells show memory responses to last for up to 8 months in COVID-19 convalescent individuals [16]. T cells that recognize SARS-CoV-2 are present in both infected and uninfected healthy exposed individuals and may play a role in protection against disease [17]. The importance of T cells in protection against SARS-CoV-2 is further reinforced by the observation of an exhausted phenotype in COVID-19 patients who are severely ill [18].

To understand the mechanisms associated with immunity against SARS-CoV-2 we compared Spike specific humoral and T cell mediated responses in different groups. Therefore, we studied a healthy control (HG) as compared with COVID-19 cases and pre-pandemic controls (PP). The HG was recruited between October 2020 and May 2021, during which time the SARS-CoV-2 circulating pandemic strains in Pakistan evolved from G clade to Alpha and then Delta variants [19–21]. We determined the presence of IgG antibodies to Spike and RBD and virus neutralizing activity of sera. Further, we measured T cell Interferon (IFN-γ) responses in using an ELISpot assay against Spike protein (S1) antigens. Hence, we compared Spike specific responses in the population prior to and during the COVID-19 pandemic, with a view to investigating factors associated with protection prior to the introduction of vaccinations.

## Methods

### Study subjects

This study was approved by the Ethical Review Committee of The Aga Khan University (AKU) (projects #2020–5152-11,688 and 2020–3687-10,181). It was conducted between October 2020 and May 2021. This was an observational study with a consecutive convenience cross-sectional sampling method.

The AKU Hospital Clinical Laboratories, Karachi, Pakistan was at the forefront of COVID-19 diagnostics from the start of the pandemic. In September 2020, the COVID-19 PCR positivity in our laboratory at Aga Khan University Hospital Clinical Laboratory, Karachi, Pakistan was 20% (unpublished data). We used this to calculate the number of samples required to investigate the prevalence of antibody positivity in this cross-sectional study. Using, a 95% CI, Z = 1.96 with 5% precision, the sample size was found to be 245. We recruited extra subjects to reduce any margin of error. We planned for healthy controls and cases in a 2:1 ratio; aiming for 300 controls and 150 COVID-19 cases. In the case of the pre-pandemic group we were limited by the availability of 114 samples present in our archives.

We invited participation from the AKU and AKU Hospital employees and their family members, sharing information regarding the study through verbal and electronic communication. Healthy participants (HG, *n* = 304) comprised 261 employees (belonging to both clinical and non-clinical areas of AKUH) and 43 of their household members. COVID-19 tests were routinely performed free of cost at AKUH for employees as part of screening protocols [22]. Thus, screening tests were conducted for 143 of the HG, all of whom tested negative by SARS-CoV-2 PCR.

All COVID-19 testing was performed by RT-PCR on nasal specimen using the Cobas 6800 SARS-CoV-2 Roche assay at the AKU Hospital Clinical Laboratories, which is accredited by the College of American Pathologists, USA.

Informed consent was taken from all study participants. Inclusion criteria were; those aged greater than 18 years, females and males, without a known history of COVID-19. At the time of recruitment, information regarding their prior clinical history was documented based on their verbal recall of any chronic viral disease such as, hepatitis B or C, HIV, immunocompromised conditions, malignancy, pregnancy or comorbid conditions.

COVID-19 cases (*n* = 163) included those aged greater than 18 years, females and males, with a SARS-CoV-2 PCR positive respiratory sample. These cases comprised COVID-19 AKUH employees, family volunteers and COVID-19 convalescent donors. The Section of Hematology, AKUH collected sera from convalescent donors between 1 and 25 weeks after their confirmed diagnosis. Patients were classified according to the WHO ordinal score [23] at the time of their diagnosis.

For testing of pre-pandemic samples, we used sera banked before the pandemic during the periods (2008–2009 and 2016–2018) as a control (PP) group. We also used banked PBMCs for the T cell ELISpot assays.

### Sample collection

Whole blood was collected for serum separation and storage at -80˚C. Sera was collected at different intervals after COVID-19 diagnosis which ranged between the same day of diagnosis up to greater than 24 weeks after diagnosis.

Blood was collected in heparin for isolation of peripheral blood mononuclear cells (PBMCs) for T cell studies from HG and COVID-19 groups. In the case of COVID-19 cases, participants were sampled within 72 h of their SARS-CoV-2 PCR based diagnosis.

### Recombinant Spike and RBD proteins

Recombinant Spike and RBD proteins were obtained from the laboratory of Prof. Paula M. Alves, iBET, Portugal. The plasmid DNA for the expression of SARS‐CoV‐2 Spike and RBD from the ancestral strains was kindly provided by Prof. Florian Krammer (Icahn School of Medicine at Mount Sinai). The soluble Spike protein presents a T4 foldon the trimerization domain, a C‐terminal hexahistidine tag and two stabilizing mutations whilst the polybasic cleavage site is absent. The soluble RBD protein includes the signal peptide and C‐terminal hexahistidine tag. Both proteins were extensively characterized and found to be both stable and consistent for use in serological assays [24].

### ELISA for IgG to Spike and RBD

All serum samples were tested at a 1/100 dilution in duplicate using an in-house enzyme-linked absorbent assay (ELISA) [25] and as per the protocol described by Figueiredo-Campos et al*.* [26]. This assay has been validated in our laboratory and described earlier [27]. Briefly, SARS-CoV-2 Spike and/or RBD protein were used to coat plates with 50 µl of Spike or RBD protein at a concentration of 2 µg/ml in PBS. Wells were blocked and then incubated with 100 µl serum samples for 2 h. Wells were washed and stained with goat anti-human IgG Fc (HRP) and then developed for colorimetric reading at 450 nm. For assay validation, sera from 45 COVID-19 convalescent cases, drawn 4 weeks after their PCR confirmed diagnosis, were used as positive controls. Sera from 55 healthy individuals from the pre-pandemic period were used as negative controls. The cut-off for positive responses of IgG to Spike and RBD was 0.5 OD450 nm in each case, by calculating the Mean + 2SD (OD at 450nm). IgG positive sera were pooled to run a dilution curve on each ELISA plate. Negative sera were also included in each ELISA run. The sensitivity of the Spike ELISA was found to be 100% (92.1–100, 95% CI) with a specificity of 100% (93.5–100, 95% CI). Sensitivity of the RBD was found to be 91.1% (78.8–97.5, 95% CI) with a specificity of 94.6% (82.4–98, 95% CI) [14, 27].

### Cell culture, virus isolation, and propagation

Vero cells (ATCC CCL-81) were cultured in DMEM media supplemented with 10% Fetal Calf Serum (FCS), 1% L-glutamine 200 mM, 1% penicillin G (100U/ml) and streptomycin (100μg/ml). Live virus was obtained from a nasopharyngeal swab (NPS) collected in viral transport medium from a PCR-confirmed SARS-CoV-2 case from June 2020. Unfortunately, we were unable to sequence this particular strain. However, we performed genomic sequencing of other isolates collected during the same week in June 2020, and showed that SARS-CoV-2 lineages L, S and G clade strains were circulating in the population [19]. Fifty microliters of serum-free DMEM were pipetted into columns 2–12 of a 96-well tissue culture plate; subsequently, 100μL of test specimens were pipetted into column 1 and serially diluted two-fold across the plate (columns 2–12; from 1 to 11 logs). Cultured Vero cells were resuspended at 1 × 10^6^ cells/mL. A hundred microliter of cell suspension was directly added to the wells of the 96-well plate containing dilutions of the clinical specimen (NPS) and mixed gently by pipetting. Inoculated cultures were grown in a humidified incubator at 37 °C with 5% CO2 for 4 days. The infected Vero cell line was observed daily for the presence of CPE, and the virus was harvested when 80%‐90% of the cells manifested CPE. The end‐point titers were calculated according to the Reed & Muench method [28] based on eight replicates for titration. The culture medium was centrifuged at 4 °C 1600 rpm for 8 min, to remove the cell debris, and then aliquoted and stored at -80 °C.

### PCR-based micro-neutralization assay

Viral neutralization was determined using our recently developed PCR-based micro-neutralization assay [29]. Serum samples were heat‐inactivated for 30 min at 56 °C. Three ten-fold serum dilutions (1:10, 1:100, and 1:1000) were prepared in media. Each serum dilution was mixed with an equal volume of live virus culture containing 100 TCID50 of SARS‐CoV‐2. The serum‐virus mixture was incubated for 1 h at 37 °C_,_ then 100μl of the mixture at each dilution was added in duplicates to a 96-well cell culture plate containing a semi-confluent Vero cell monolayer then incubated for 24 h. Cells without virus served as ‘cell line control’, while cells with the virus without serum served as ‘virus control’.

After incubation of 96-well plates for 24 h, the supernatant was carefully removed, and cells were washed with DMEM media. RNA extracted from cells and then used to perform a rapid real-time PCR using Novel Coronavirus (2019-nCOV) Nucleic Acid Diagnostic Kit (PCR Fluorescence Probing) of Sansure Biotech (S3102E) (Changsha, China) as described before [30]. The assay sensitivity of Sansure Biotech is 1000 copies/ml. This was validated in our laboratory to detect SARS-CoV-2 viral RNA with a Ct cut-off of 39. For 2019-nCoV-PCR, a negative result was defined as Ct value ≥ 40, while positive control was defined as Ct value No ≤ 35, as per kit’s instructions [31].

The SARS-CoV-2 PCR Ct values obtained for each serum-virus well, and control wells containing cells alone and virus control, were averaged for each sample. The average Ct values obtained were used to measure the percent inhibition/neutralization using the formula [32]: 100 − ((*N*‐average Ct of ʻcell line control’ wells)/(average Ct of ʻvirus controlʼ wells‐average of ʻ cell line control’ wells)*100), where *N* is the average Ct for each well/sample.

### T cell ELISpot assay to Spike antigens

T cell responses to SARS-CoV-2 were assessed using ex vivo method of IFN-γ ELISpot Mabtech, AB, Sweden. PBMCs were plated at 250,000/well in a 96 well plate Cells were stimulated in duplicates with Spike S1 antigen from an ancestral strain (Peptivator S1 (Miltenyi, Biotec) at 4μl/well). Negative controls lacked any peptide stimulation. Positive controls were set up using human T cell activator CD3/CD28 (Gibco). The assay was performed as per the manufacturer’s instructions. Spots were captured using a USB microscope and counted. Mean values were calculated from duplicate wells set per sample. Results for each condition were obtained by subtraction of values of the test from ‘nil’ wells without stimulation.

### Statistical analysis

The normality of data was checked through Shapiro–Wilk test. The IgG antibody data for the study groups (HG, COVID-19 and PP) did not have a normal distribution (alpha *p* value was < 0.05). The median (IQR) was calculated as for skewed continuous data. Frequency or proportion was used to give estimates for categorical data. The Kruskal–Wallis test were used for comparison of non-parametric data between groups. Correlation between the Spike and RBD antibody levels was determined using the Spearman’s rank correlation test using the GraphPad prism. A *p*-value ≤ 0.05 was considered significant.

## Results

### Characteristics of the study subjects

Study participants were recruited between October 2020 and May 2021. We recruited 304 HG and 163 COVID-19 cases. To investigate the prevalence of IgG antibodies in the pre-pandemic period we studied banked sera collected prior to 2018 as the PP group. Age and gender of individuals from all three groups is also shown in Table 1. There was no significant difference between the age and gender of the HG, COVID-19 and PP groups.

**Table 1:** Demographics and clinical characteristics of study subjects

	**HG**	**COVID-19**	**PP**
Demographics			
Number of subjects (n)	304	163	114
Age (years)	30.34 ± 12.02	34.73 ± 2.82	42.8. ± 14.7
Sex (% females)	59.2	35.5	49.7
Clinical Characteristics			
Fever/chills (%)	-	47.23	-
Cough (%)	-	27.60	-
Sore throat (%)	-	21.47	-
Shortness of breath (%)	-	10.42	-
loss of taste/ smell (%)	-	0.61	-
Comorbid			
Diabetes (%)	0.01	3.06	-
Hypertension (%)	0.02	3.06	-
Asthma (%)	0.02	0.61	-
Others^a^ (%)	1.97	5.52	

*HG* Healthy control Group (*n* = 304); COVID-19 (*n* = 163), *PP* pre-pandemic controls (*n* = 114). Data are presented as numbers (n), percentage (%) or median (interquartile range), unless otherwise stated. Others^a^ denote comorbid conditions including individuals with hypothyroidism, Grave's disease, Celiac disease, hyperlipidemia and thalassemia minor. Statistical analysis between groups was performed using the Kruskal Wallis test, *p* ≤ 0.05 was considered significant, ‘ns’ not significant

### Seroprevalence of IgG to Spike and RBD protein in HG, COVID-19 cases and PP groups

**Fig. 1:**
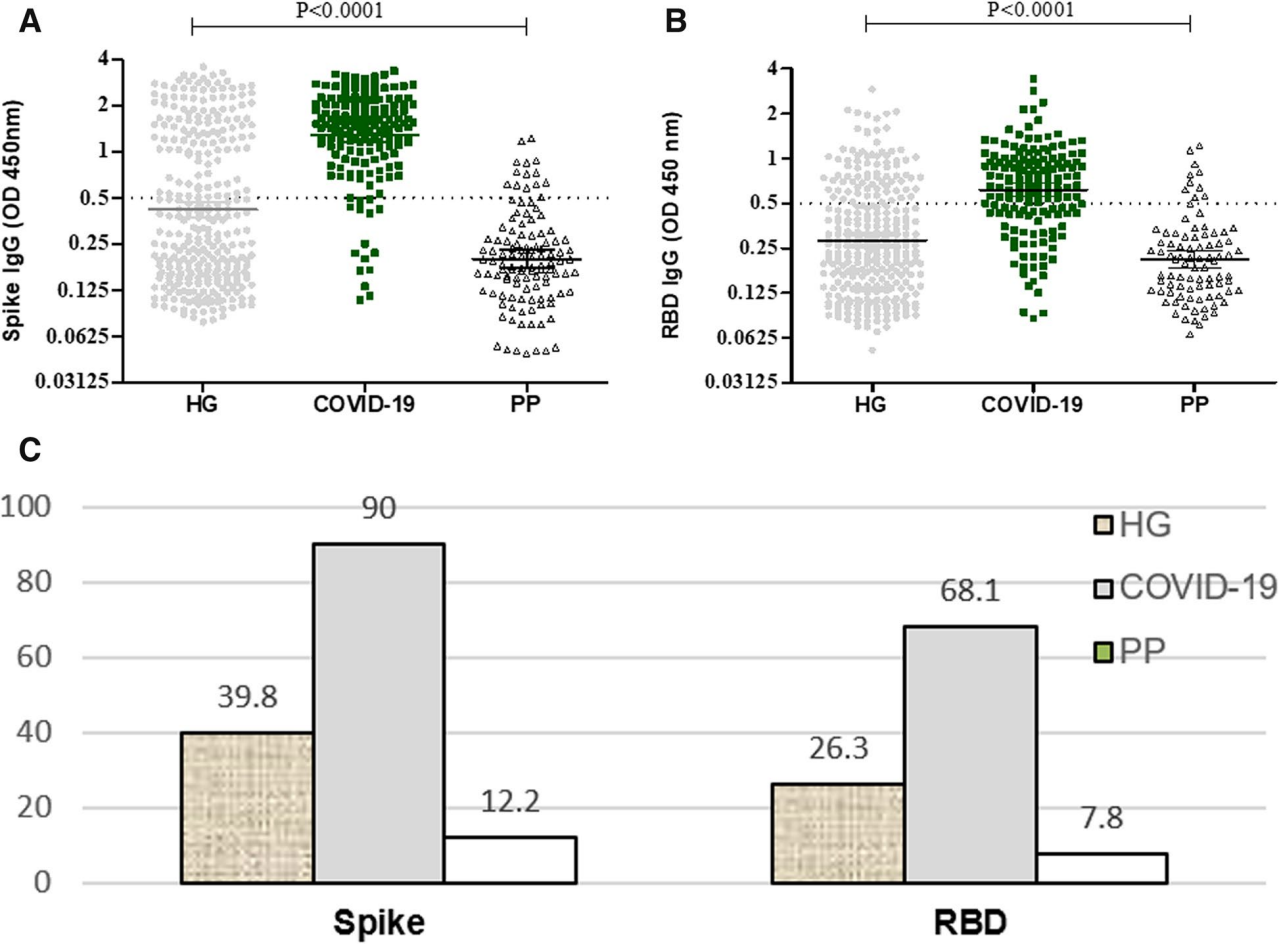
IgG antibody levels to Spike and RBD are detected in the Healthy Group (HG), COVID-19 and PP groups. IgG antibodies were determined in sera of HG (*n* = 304), COVID-19 cases (*n* = 163) and PP (*n* = 114). Graphs show IgG levels to **A**, Spike and **B**, RBD. The cut-off for positive responses at 0.5 OD 450 nm is indicated by a dotted horizontal line. Data is shown with geometrical mean indicated by horizontal line and 95%CI as error bars. The Kruskal–Wallis test was used to calculate the statistically significant differences (*p* ≤ 0.05) between groups. **C** The frequency of individuals who are IgG seropositive to Spike and RBD is shown

We measured IgG antibodies to Spike and RBD in sera of HG, COVID-19 and PP groups. Anti-Spike IgG levels were found to differ between groups, with higher antibody levels in HG and COVID-19 cases as compared with PP (*p* < 0.0001, Kruskal–Wallis test) cases, Fig. 1A. IgG antibodies to RBD in the HG, COVID-19 and PP groups were found to be significantly different (*p* < 0.0001). IgG levels were raised in HG and COVID-19 cases as compared with PP, Fig. 1B. The IgG seropositivity of each group was determined. Seropositivity to Spike was highest in COVID-19 cases at 90%, followed by 39.8% in HG and 12.2% in PP groups, Fig. 1B. Similarly, seropositivity to RBD was highest in COVID-19 cases at 68.1%, followed by 26.3% in HG and 7.8% in PP groups, Fig. 1C.

### Correlation between IgG to Spike and RBD in HG, COVID-19 and PP

**Fig. 2:**
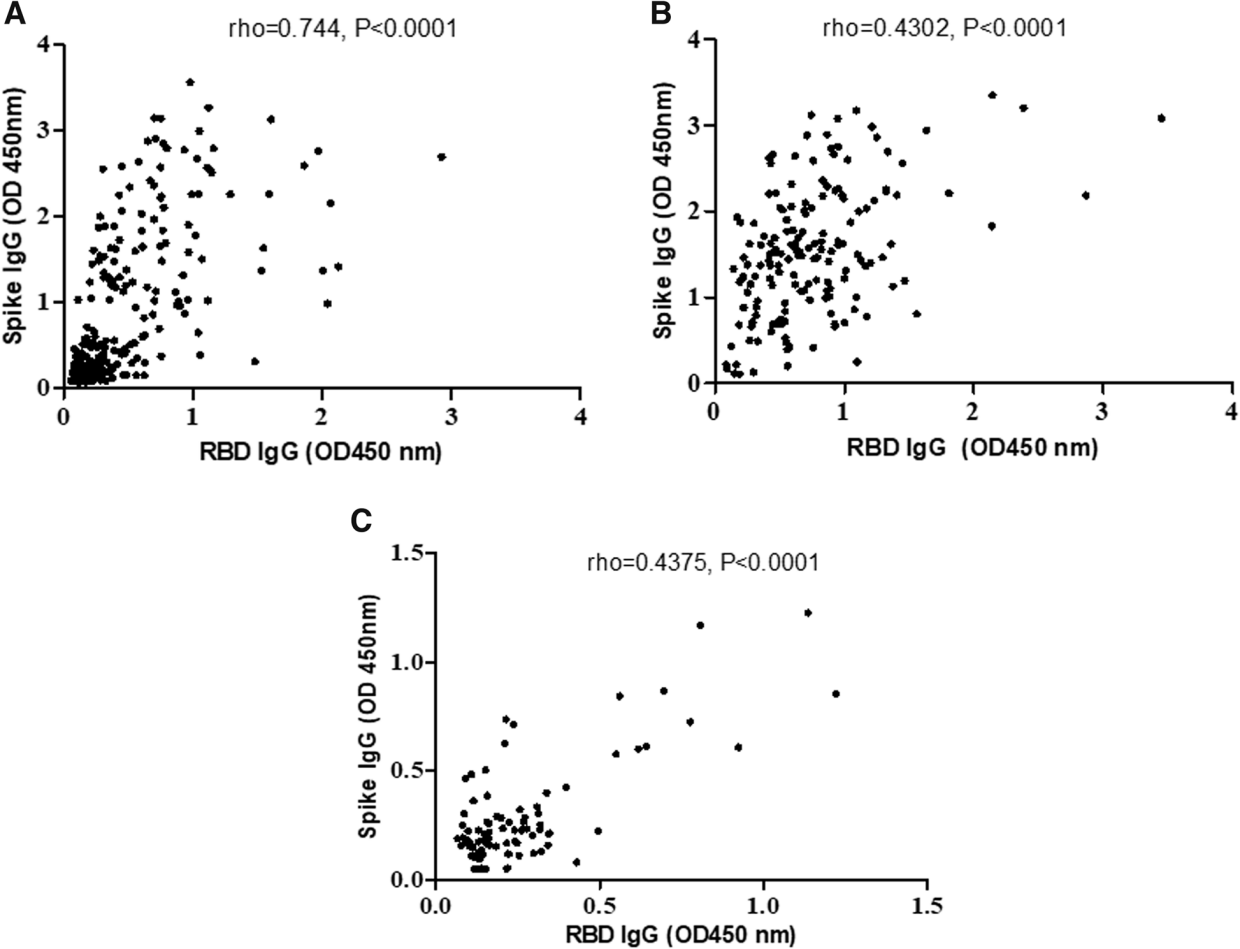
Correlation of IgG antibody levels to Spike and RBD in HG, COVID-19 and PP groups. IgG levels to Spike and RBD in HG, COVID-19 and PP cases were analysed using a Spearman’s rank correlation analysis. The correlation (rho) and significance (p) are indicated on the figure panels for **A**. HG, **B**. COVID-19 and **C**. PP groups

We determined the correlation coefficient between IgG antibodies to Spike and RBD proteins in the study groups using the Spearman’s Rank analysis. A significant correlation was observed between IgG to Spike and RBD in the HG (Fig. 2A, SR rho = 0.744, *p* < 0.0001), in COVID-19 cases (Fig. 2B, SR rho = 0.430, *p* < 0.0001) and in PP (Fig. 2C, SR rho = 0.438, *p* < 0.0001). Showing, a positive association between IgG antibodies to Spike and RBD in all three groups. Of note, the slope of the SR curve differed between the study groups, with the strongest in COVID-19 cases. This suggests recognition of common antigens by the antibodies in each of the groups. The slope of the HG and COVID-19 groups denoted higher IgG levels to Spike. The lowest correlation rank was observed in the case of PP, where the antibody levels to Spike were lower than in HG and COVID-19 groups. Antibody responses may vary according to the time after exposure and also the severity of illness. To examine this further, we interrogated the IgG antibody levels in HG across the study period and also, investigated antibody levels in COVID-19 cases with active or recovered disease.

### Time-course analysis of IgG antibody levels in healthy individuals

**Fig. 3:**
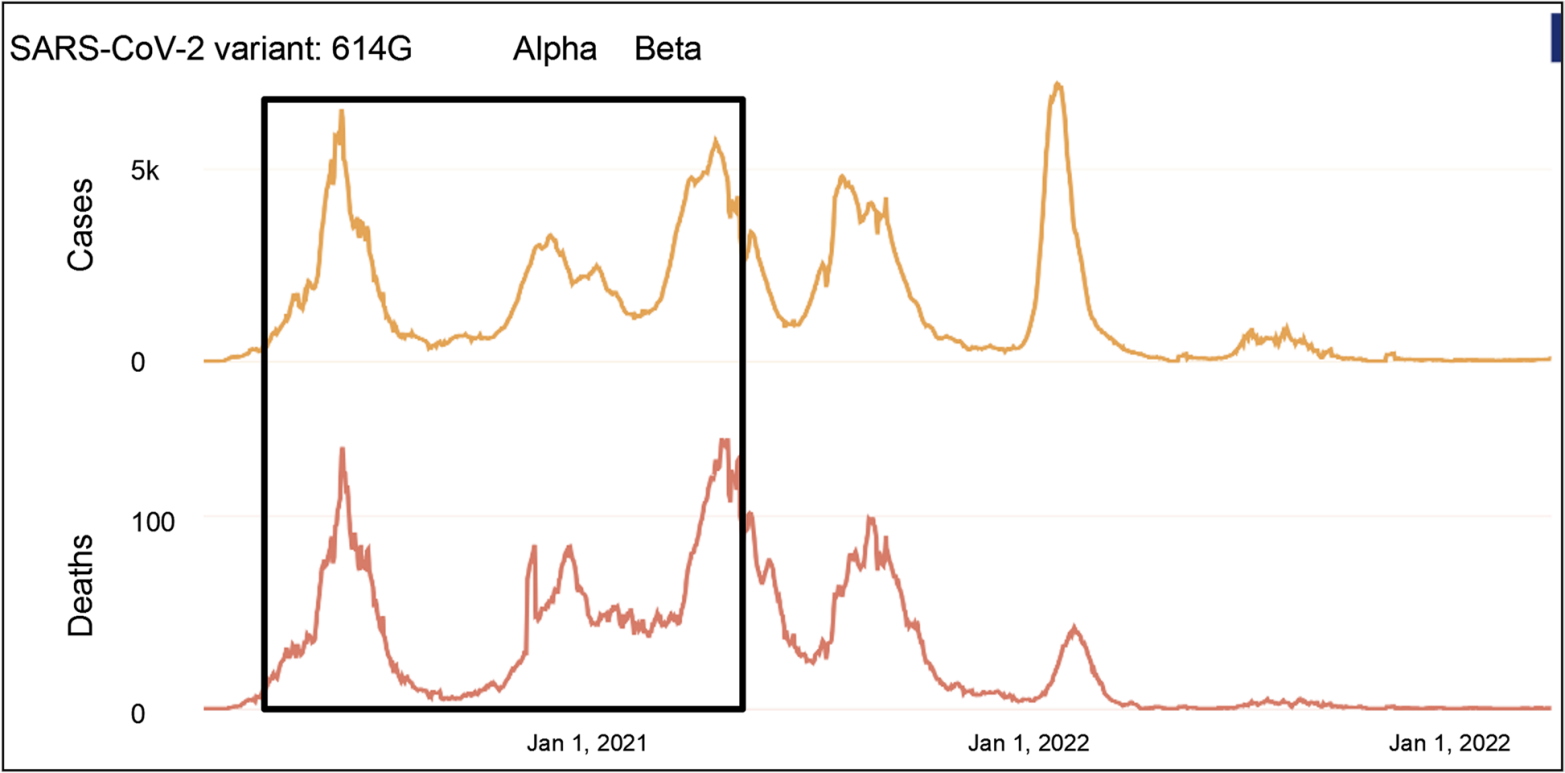
Time-line of the COVID-19 pandemic in Pakistan. Data presented is for the COVID-19 positive cases (upper panel) and deaths (lower panel) in Pakistan between months March 2020 and till February 2023. Source, John Hopkins, Corona Research Center https://coronavirus.jhu.edu/region/pakistan. The boxed region defines the period of this study. SARS-CoV-2 variants associated with the waves seen during the period are listed

Pakistan experienced three COVID-19 waves during the study period October 2020 until May 2021 (Fig. 3). The wave in October 2020 was associated with mainly GH and GR clade strains [19], with the introduction of Alpha variants in January 2021 [20] and Beta variants in March/April 2021 [21]. We examined IgG antibody responses of healthy individuals (HG) sampled across the study period, subsequently analysed in two monthly intervals; Oct-Nov’20 (*n* = 66), Dec’20-Jan’21 (*n* = 65), Feb-Mar’21 (*n* = 155) and Apr-May’21 (*n* = 18).

**Fig. 4:**
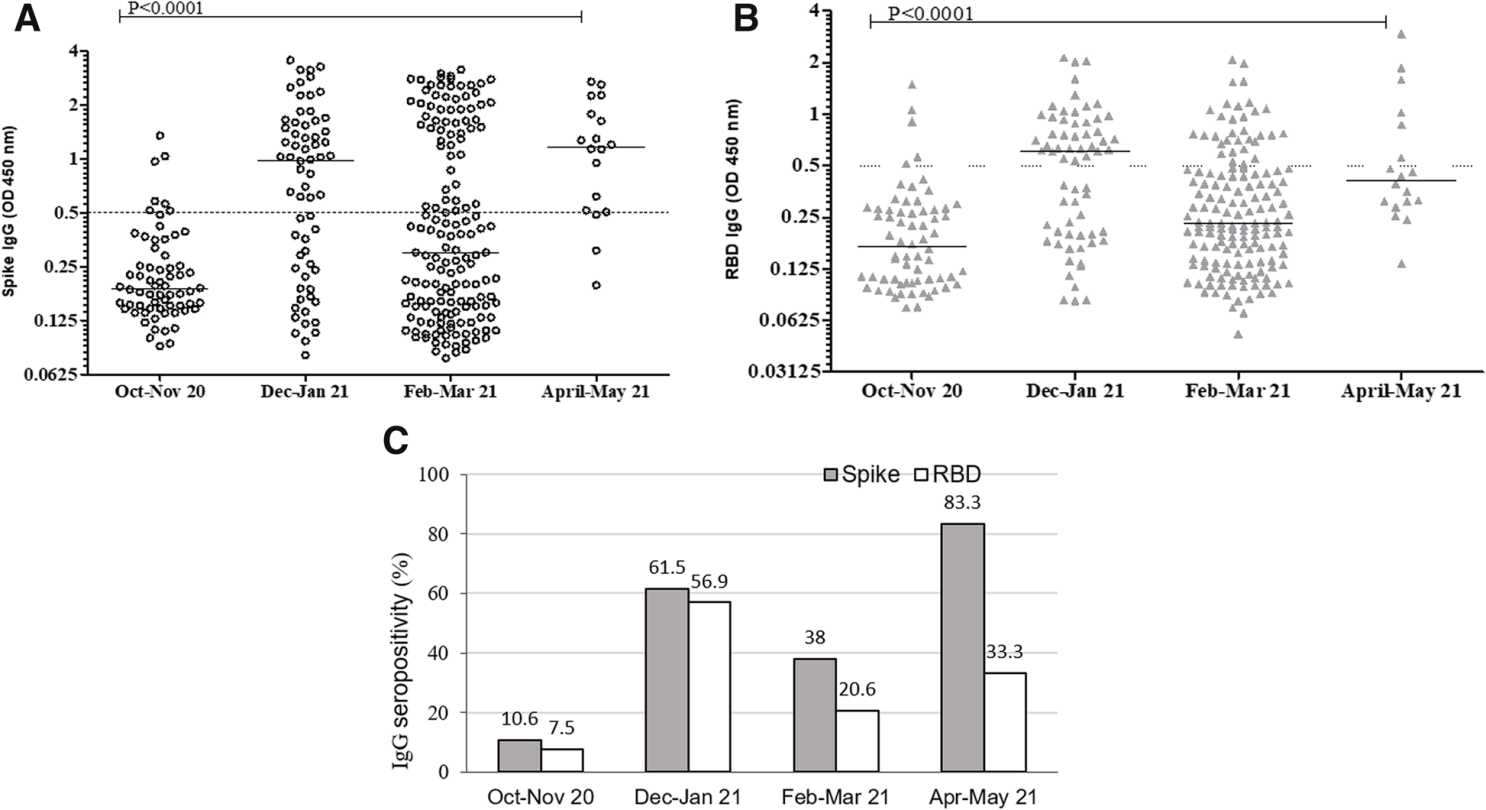
Time-course analysis of IgG antibody levels to Spike and RBD in a Healthy Group. IgG antibodies in the healthy group (HG) were analysed in two-monthly intervals between October 2020 to May 2021. Antibody levels in participants are shown for Oct-Nov’20 (*n* = 66), Dec’20-Jan’21 (*n* = 65), Feb-Mar’21 (*n* = 155) and Apr-May’21 (*n* = 18). IgG levels to Spike (**A**) and RBD (**B**) are depicted. Data are shown as geometric means as horizontal line and 95%CI as error bars. The positive cut-off (0.5 OD) is shown by a dotted line. **C** The frequency of seropositive responses to Spike and RBD in each period is shown. Significant differences (*p* ≤ 0.05) calculated by MWU analysis

There was a significant increase in IgG levels to Spike (Fig. 4A, Kruskal–wallis, *p* < 0.0001) and RBD (Fig. 4B, p < 0.0001) across the study period. IgG levels to Spike in HG recruited in Oct-Nov 2020 were significantly lower than those in Dec20-Jan21 (*p* < 0.0001), Feb-Mar21 (*p* = 0.036) and Apr-May21 (*p* < 0.0001). Similarly, IgG levels to RBD were lower in Oct-Nov20 were lower than those observed in Dec20-Jan21 (*p* < 0.0001), Feb-Mar21 (*p* = 0.00018) and Apr-May21 (*p* < 0.0001). The IgG antibodies were also assessed as seropositivity and the frequency of IgG to Spike was seen to increase from 10.6% in Oct-Nov20 to 83.3% in Apr-May21 (Fig. 4C). The percentage of individuals seropositive to RBD increased from 7.5% in Oct-Nov to 33.3% in Apr-May21, respectively.

### Temporal association of IgG to SARS-CoV-2 in COVID-19 cases

**Fig. 5:**
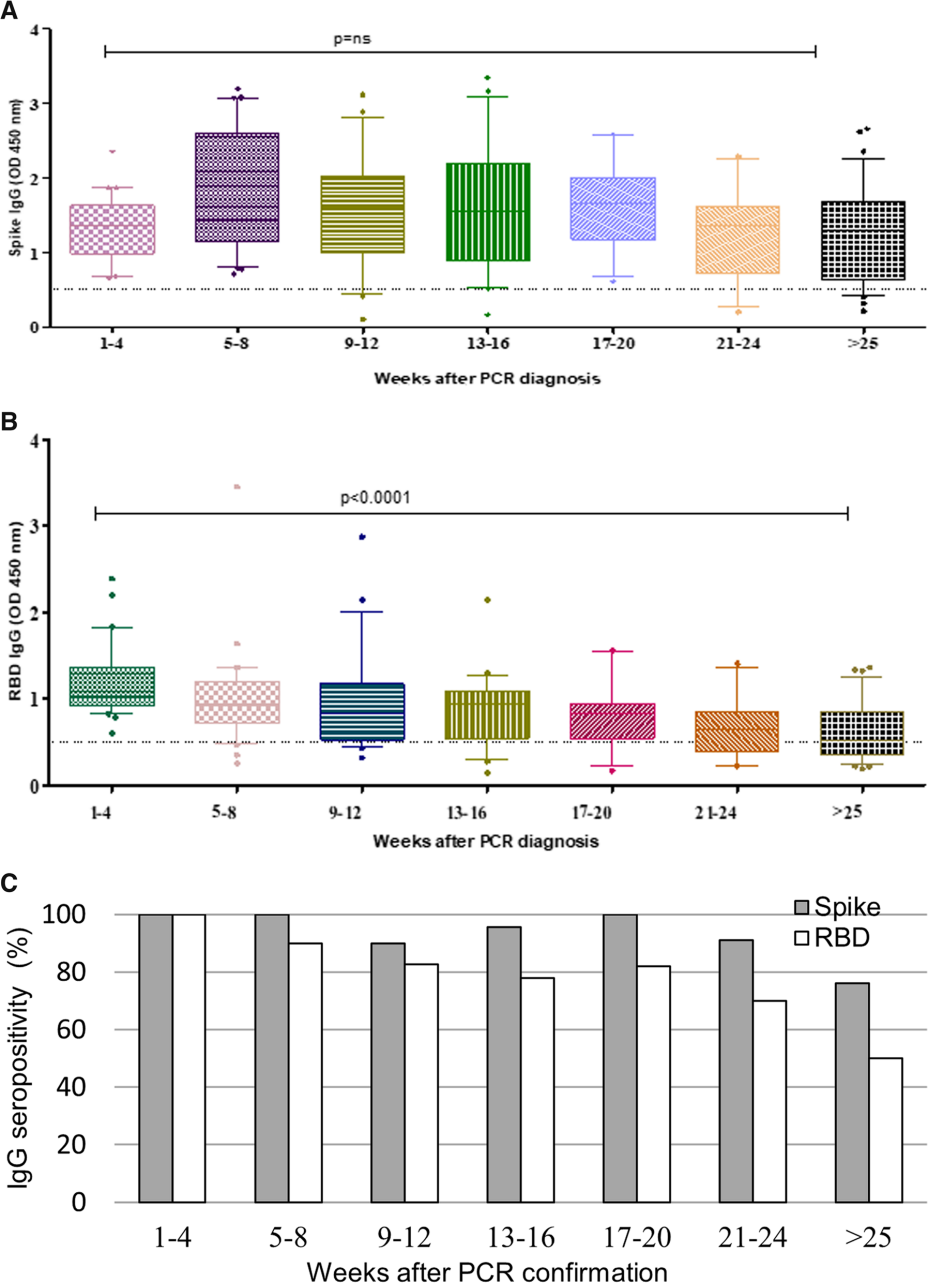
Temporal association of IgG to SARS-CoV-2 antigens in COVID 19 cases. Levels of IgG antibodies were assessed in COVID-19 cases over a period of time (1 to > 25 weeks) post-diagnosis. Data is depicted in the form of scatter plot with horizontal line indicating the median values. Cut-off of positivity was considered to be OD value ≥ 0.5 indicated by a dotted line. Graphs how IgG levels to (**A**) Spike, and (**B**) RBD in COVID-19 cases; (**C**) Percentage of samples positive for IgG to Spike and RBD recruited over a period of 6 months

The majority of COVID-19 cases (*n* = 157) had minimal disease, ranked as WHO Ordinal score of 1 or 2. Six COVID-19 cases (3.6%) required hospitalization and had COVID-19 ordinal scores of 3 (*n* = 3), 4 (*n* = 2), and 5 (*n* = 1), respectively at admission. All symptomatic COVID-19 cases made a full recovery. We further defined the levels of IgG to Spike and RBD in the patients as per the time their sample was collected after their diagnostic PCR test (between 1 and > 25 weeks). IgG to Spike positive responses were detectable within a week of SARS-CoV-2 PCR diagnosis, with no significant difference in antibody levels over the study period (*p* value, not significant, Fig. 5A). However, whilst IgG to RBD was also detectable in sera of COVID-19 within the first week after diagnosis, we found a decrease in IgG levels to RBD over the following period (*p* < 0.0001, Fig. 5B). The frequency of seropositive responses to both antigens was determined. All COVID-19 cases were seropositive to Spike between 1 and 8 weeks post-diagnosis then varied between; 100 and 90% seropositivity was found in those tested between 12 and 20 weeks after their diagnosis; 91% positivity up to 24 weeks and reducing to 76% in those tested after 24 weeks, Fig. 5C. IgG seropositivity to RBD was present in all cases at 4 weeks, 90% at 8 weeks, 82.6% at 12 weeks, 78% and 82% at 16 and 20 weeks respectively, followed by a reduction to 50% in those measured after 24 weeks of COVID-19 diagnosis.

### Virus neutralizing ability of IgG antibodies to RBD

**Fig. 6:**
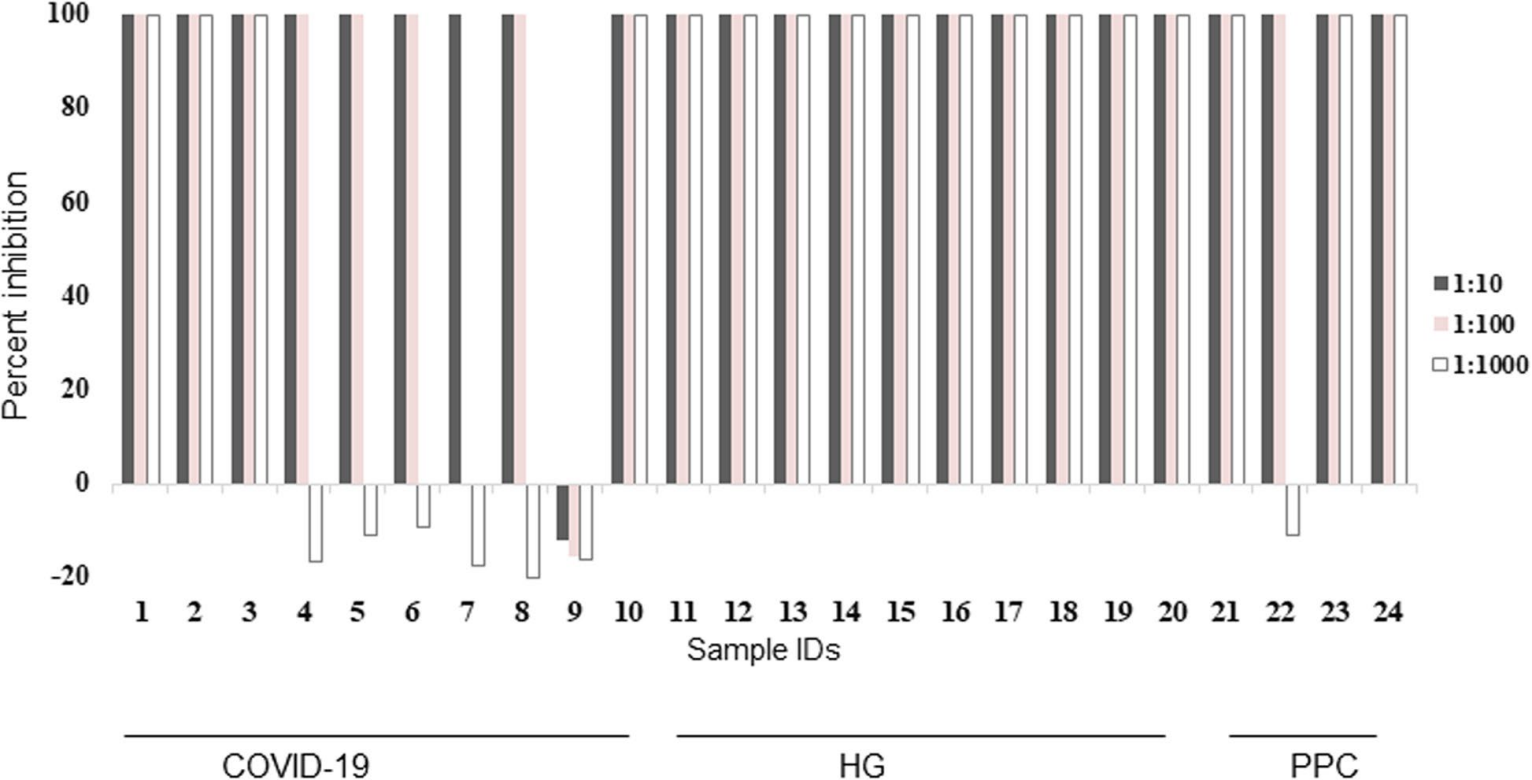
Neutralization activity for IgG positive serum samples. Virus neutralization potential of the serum with antibodies to RBD was tested at three different serum dilutions (1:10, 1:100, 1:1000) using a PCR based microneutralization assay. Sera of COVID-19 cases (S1-10), HG (S11-20) and PP (S21-24) were tested. The percentage of inhibition is calculated based on the amplification of SARS-CoV-2 RNA present by RT-PCR (Ct) compared with a control specimen (0%) in each case

Antibody levels usually reflect biological potency such as neutralizing activity. We used a PCR-based virus micro-neutralization assay established in our laboratory [33] to investigate the virus-neutralizing potential of the IgG antibodies observed in study subjects. Twenty four sera from HG (*n* = 10), COVID-19 (*n* = 10) and PP (*n* = 4) which had been previously tested for IgG antibodies to both Spike and RBD were used. SARS-CoV-2 neutralizing activity was found in IgG positive sera of the HG, COVID-19 and PP cohorts. Of these, 18 showed complete neutralizing potential against SARS-CoV-2 including, three amongst COVID-19, 10 from HG and three from PP groups, Fig. 6. Five sera only partially neutralized SARS-CoV-2 infection of Vero cells (four from COVID-19 and one from PP). One serum sample from the PP group did not show any neutralizing activity.

### Reactive T cells secreting Interferon-gamma in COVID-19 cases, HG and PP groups

**Fig. 7:**
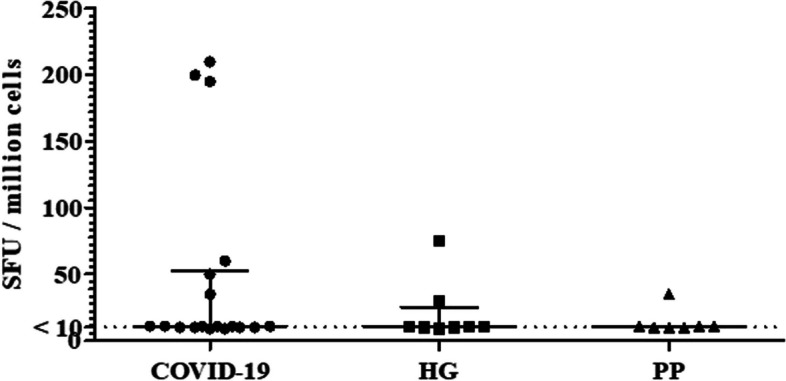
T cell IFN-γ activation observed in response to SARS-CoV-2 Spike protein in COVID-19 cases, healthy individuals from the pandemic and pre-pandemic period. The presence of reactive T cells secreting IFN-γ to Spike S1 protein was determined by ELISpot analysis. PBMCS were plated on IFN-γ coated plates and stimulated with S1 protein for 18 h. The graph depicts the number of T cells secreting IFN-γ identified for COVID-19 (*n* = 18), HG (*n* = 6) and PP (*n* = 7) groups

We investigated whether T cells recognizing Spike antigen were present using an ELISpot assay to identify cells reactive to S1 ancestral antigen. We found that each of the study groups had individuals with T cells reactive to Spike. Amongst COVID-19 cases, six of 18 (33.3%) individuals had T cell reactivity to S1 antigen, Fig. 7. T cell recognition was observed in two of seven (28.6%) of HG and one of six (16.7%) of PP study subjects.

We next determined whether there was any relationship between T cell recognition and IgG antibody responses observed. Of the 18 COVID-19 cases, six had IgG antibodies to Spike but only one had IgG antibodies to RBD. None of the seven HG for whom ELISpot assays was performed had positive IgG antibodies to Spike or RBD. Of the seven PP controls, one individual had IgG antibodies to both Spike (OD 0.608) and IgG to RBD (OD 0.924). We further divided the COVID-19, HG and PP groups into those who were SARS-CoV-2 T cell reactive and those who were not. Supplementary Fig. 1 depicts Spike IgG antibodies in individuals of each groups based on whether they were T cell reactive ( +) or T cell negative (-). Five COVID-19 cases had IgG antibodies to both Spike and RBD, three of whom had SARS-CoV-2 reactive T cells and two who did not. Of the HG group, none had IgG to Spike including, the individual with a positive T cell IFN-γ response. Of the PP group, the individual with a positive T cell responses did not have IgG to Spike or RBD. These data showed that IgG antibodies to Spike protein were independent of T cell responses in the groups studied.

## Discussion

Our study of IgG antibodies to Spike and RBD protein accompanied by neutralizing assays against SARS-CoV-2 and determination of T cell IFN-γ responses to SARS-CoV-2 antigens identifies the presence of humoral and T cell responses that protect against viral infection. Identification of IgG antibodies to Spike and RBD, neutralizing activity and T cell reactivity to Spike in pre-pandemic controls indicates that immunity against SARS-CoV-2 was present in the population prior to the COVID-19 pandemic. The increasing levels of IgG antibodies in healthy controls tested across the study period October 2020 until May 2021 are reflective of the adaptation of humoral responses in the population likely, due to increased exposure to SARS-CoV-2 even in uninfected healthy controls.

Overall, we found that 39.8% of individuals were reactive to Spike and 26.3% to RBD in the HG group while, 90% of COVID-19 case were reactive to Spike and 68.1% to RBD.

We studied the changes in antibody responses with time after COVID-19 diagnosis, testing individuals within 1 week and up to greater than 24 weeks afterwards. We observed that Spike IgG levels and seropositivity did not change much up until 24 weeks but that there was a reduction after 25 weeks or more. The lower levels of RBD antibodies found by 24 weeks after COVID-19 infection indicates a waning of the elicited response. Our subjects primarily had asymptomatic/minimal disease and therefore we are unable to comment on comparisons between antibody responses between asymptomatic and symptomatic COVID-19. Not all antibodies are neutralizing as was demonstrated by our sub-set of samples tested in the neutralizing antibody assay.

Interestingly, 12.2% of pre-pandemic controls tested were found to be seropositive to Spike and 7.8% were seropositive to RBD. IgG antibodies to Spike and RBD in sera collected prior to the COVID-19 epidemic suggests the presence of cross-reactive antibodies. There was a positive correlation between IgG antibodies to Spike and RBD in sera of HG, COVID-19 and PP groups. Indicating, the recognition of similar but distinct epitopes in these groups. The lower magnitude of IgG titers to Spike and RBD found in the pre-pandemic group are in line with previous observations that cross-reactive antibodies have lower affinity to SARS-CoV-2 antigens than those induced by SARS-CoV-2 infection [34]. Cross-reactive antibody responses to SARS-CoV-2 have been observed in the context of seasonal human coronavirus (HCoVs) infections [21–23]. Global studies reveal cross-reactivity to other coronaviruses to be present in Sub-Saharan Africa [35], and Kenya [36]. Further, cross-reactive SARS-CoV-2 antibodies were identified in pre-pandemic sera in Italy [37]. Whilst the nature of cross-reactive responses may vary, they have been shown to enhance humoral and cellular responses against SARS-CoV-2 [24, 25].

The assessment of the duration of IgG antibodies is important as it plays a crucial role in early protection against the disease as well as during re-infection [38]. IgG antibodies induced by SARS-CoV-2 infection are believed to last up to 10 months or more [39]. Waning of immunity against SARS-CoV-2 has been shown to occur with time [40]. The increase in IgG seropositivity we observed between October 2020 and May 2021 in healthy individuals is consistent with increasing seroprevalence in the population through the pandemic. Seropositivity to Spike rose from 10% in October to 83% in May 2021. The rising IgG positivity fits with the study period which includes the second (October 2020 until January 2021) and third (April until May 2021) waves of the pandemic respectively [8]. This period was characterized by a change in circulating strains from the ancestral Wuhan, S and L clades to G clade strains by the latter part of 2020 followed by the introduction of Alpha variants in January 2021, followed by Beta variants [19–21]. The rising seropositivity correlates with data from seroprevalence studies conducted in Karachi during the early pandemic period whereby, Zaidi *et* al. showed that between April and July 2020, COVID-19 seropositivity varied between industrial employees (50%), community (34%) and healthcare workers in Karachi (13%) [41]. Batool *et* al. showed showed COVID-19 seroprevalence to be 33% in healthcare workers across Pakistan [42]. Further, serial population based serosurveys for COVID-19 in Karachi neighbourhoods showed a rise from 0.2% to 12.8% seropositivity in low transmission-areas, and a rise from 0.4% to 21.8% in high transmission areas between April and August 2020 [43]. Global data indicates higher seroprevalence to SARS-CoV-2 in healthcare workers as compared with the general population [44].

Importantly, we only tested unvaccinated individuals and therefore seroprevalence is attributable either to pre-existing immunity or due to exposure and sub-clinical infection with SARS-CoV-2. Of note, seroprevalence studies conducted do note that a predominant proportion of individuals with antibodies against SARS-CoV-2 were asymptomatic [41–43].

Examination of sera from COVID-19 cases revealed high positivity to SARS-CoV-2 (90% to Spike, 68.1% to RBD). IgG to Spike remained present in individuals who were recruited up to greater than 24 weeks after their COVID-19 diagnosis. Whilst, IgG to RBD was found to wane after 8 weeks. The waning of immunity against SARS-CoV-2 has been shown to occur with time [40].

Antibody responses have also been used to follow immunity due to vaccination strategies whereby, IgG to RBD are associated with neutralizing activity to SARS-CoV-2 as a measure of successful COVID-19 vaccination [45].

Virus neutralizing assays demonstrate the ability of IgG to block entry into host cells [46]. We observed neutralizing activity of sera to be associated with IgG to RBD in HG, COVID-19 and PP. The presence of neutralizing antibodies in COVID-19 convalescent cases are in line with protection imparted by activation of B cells in response to COVID-19 infection [16]. Reports on the activity of cross-reactive sera can be contradictory whereby, some do not associate them with neutralizing activity against SARS-CoV-2 [47]. However, others have reported neutralizing antibodies which are cross-reactive to the Nucleocapsid protein [48]. Further, Ng, et al. showed that cross-reactive sera to Spike and Nucleocapsid protein had neutralizing activity [49].

Cellular immunity driven by T cells is important for protection against SARS-CoV-2 [50]. We found T cells reactive to Spike protein antigen to be present in HG, COVID-19 and PP groups. Of the COVID-19 cases, 33% of individuals had reactive T cells. The absence of the same in the remaining subjects could be due to early collection of samples within 48 – 72 h of COVID-19 diagnosis. T cell depletion has been observed in COVID-19 patients, with a progressive reduction in CD4 and CD8 T cells in those with severe infection as compared with mild COVID-19 disease [15]. The presence of reactive T cells in the HG group is supported by earlier reports, which show cellular immunity to SARS-CoV-2 in individuals exposed to SARS-CoV-2 but who are uninfected [17]. Amongst the pre-pandemic controls, one of six individuals had positive T cell response to Spike S1 protein. This reflects heterogeneity in the pre-pandemic population.

Previous reports have shown T cell responses against SARS-CoV-2 in healthy unexposed individuals to be between 28 to 50%, while pre-existing immunity has been shown in multiple samples stored in pre-pandemic period [51, 52]. Further, cellular immunity also plays a role as pre-existing T cell immunity to hCoVs can also prime responses against SARS-CoV-2 [53].

Appropriate activation of T cell responses leaves behind memory T cells [16]. Long lasting memory responses are essential for protection such as, in case of re-infections and it is thought that the endemic immunity will maintain protection against SARS-CoV-2 even after the pandemic [54].

When T cell reactivity was correlated with the presence of IgG antibodies, we found that these were independent of one another. B cells, NK cells and gamma delta T cells may be activated independently of T cell responses and then play a role in modifying adaptive immunity. Cross-reactive antibodies present in circulation likely precede the higher affinity antibodies produced by plasma cells from germinal centers as a consequence of viral infection [55].

There is limited information regarding circulating hCoVs in the country. It is possible that cross-protection to SARS-CoV-2 could be induced by hCoVs in addition to other factors. An expansion of responses against unrelated pathogens is seen in the case of infection with *Mycobacterium tuberculosis* resulting in antibody recall responses to respiratory tract pathogens such as, respiratory syncytial virus and measles virus [56].

Serological studies conducted in countries endemic to TB have shown to have higher seroprevalence to SARS-CoV-2 as compared to the non-TB endemic region [35]. BCG has been shown to be associated with recall or memory responses that protection against non-related viruses and bacteria [57, 58]. However, the impact of BCG related to COVID-19 is still under discussion [59].

A limitation of our study is that we could not confirm the source of cross-reactive antibodies in our groups by testing for reactivity to antigens such as those of seasonal coronaviruses. Another limitation is that we do not have longitudinal sampling for either the HG or the COVID-19 cases. Unfortunately, study subjects were unwilling to give follow up specimens. Due to hesitancy during the study period including, anxiety about coming to the research laboratory to submit blood samples.

Therefore, our data primarily depicts the occurrence of IgG antibodies in different unvaccinated, healthy individuals with no known history of COVID-19 who enrolled across the study period. However, the dynamics of IgG responses to Spike and RBD we observed in the HG group matches well with observations of seroprevalence in other studies [41, 43] in the context of a group exposed to COVID-19 waves. We used a PCR based microneutralization assay which was developed in our laboratory [33]. Unfortunately, we could only conduct neutralizing assays on a limited number of samples due to technical limitations and difficulty in accessing the required reagents and cell lines.

## Conclusions

Overall, this study indicates the presence of antibodies and T cells that recognize SARS-CoV-2 in the pre-pandemic population and also in healthy individuals through the COVID-19 pandemic. In the context Pakistan, it is possible that the relatively lower morbidity and mortality from COVID-19 observed may be due to factors such as, other infections which have led to cross-protective immunity against SARS-CoV-2 [14, 35, 60]. IgG antibodies to RBD are associated with neutralizing activity to SARS-CoV-2, and as a measure of successful COVID-19 vaccination [44]. Further, pre-existing SARS-CoV-2 immunity influences the potency and durability of the response to vaccination [61]. Hybrid immunity that results from a combination of prior COVID-19 infection and vaccination is believed to play an important role in the maintenance of immune protection. Increased immunity against SARS-CoV-2 in the Pakistani populations has significant policy implications for the continued roll-out of vaccination and booster strategies thereby impacting healthcare costs and effort.

### Supplementary Information

**Additional file 1: Supplementary Figure 1.** IgG antibodies to Spike protein are independent of T cell responses in COVID-19 cases and controls. The IgG antibody results (OD values) against RBD in each group are shown in COVID-19, HG and PP cases which are listed as ‘-ve’ or ‘+ve’ based on the presence of absence of T cells as determined by ELISpot analysis.

## Data Availability

The datasets used and/or analysed during the current study are available from the corresponding author on reasonable request.
