# Supplementary material for: Humoral and T cell responses to SARS-CoV-2 reveal insights into immunity during the early pandemic period in Pakistan
Source: BMC Infect Dis. 2023 Dec 1;23:846. doi: 10.1186/s12879-023-08829-1 (PMC10691108; doi:10.1186/s12879-023-08829-1)

Supplementary Figure 1. IgG antibodies to Spike protein are independent of T cell responses in COVID-19 cases and controls. The IgG antibody results (OD values) against RBD in each group are shown in COVID-19, HG and PP cases which are listed as '-ve' or '+ve' based on the presence of absence of T cells as determined by ELISpot analysis.

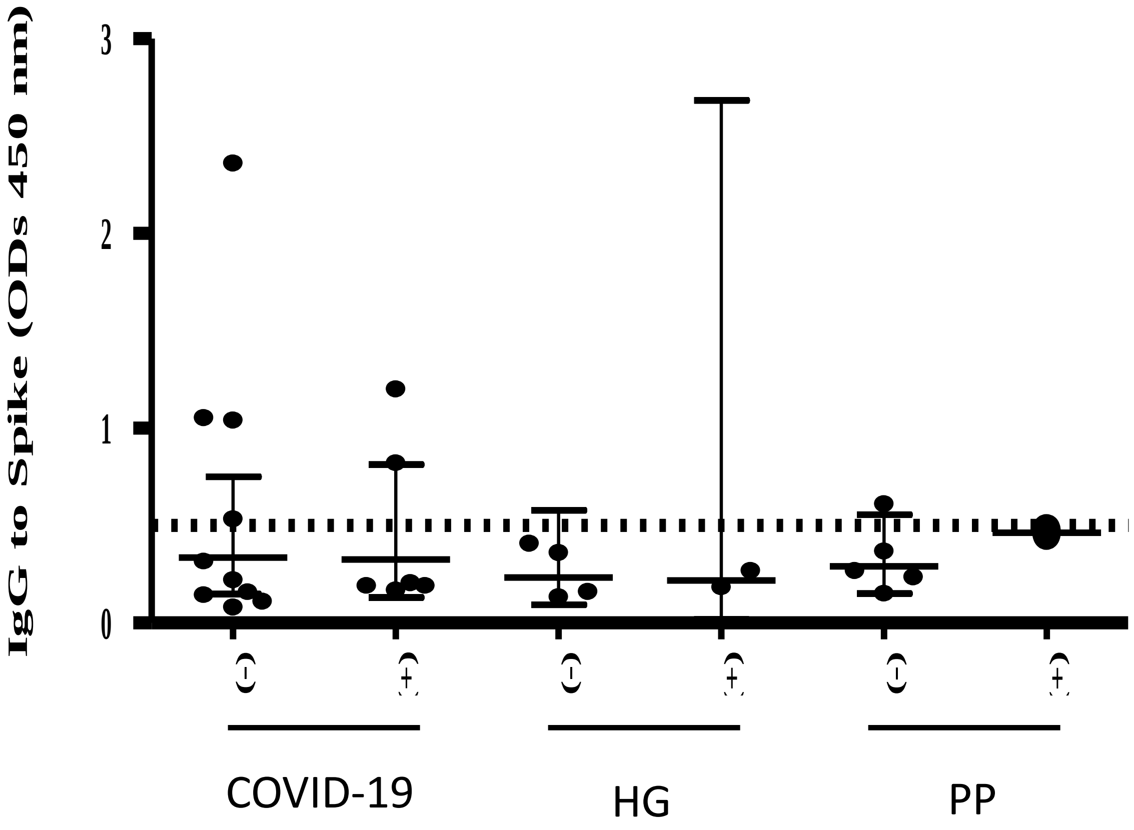

Supplement: Supplementary file 1 — Additional file 1: Supplementary Figure 1. IgG antibodies to Spike protein are independent of T cell responses in COVID-19 cases and controls. The IgG antibody results (OD values) against RBD in each group are shown in COVID-19, HG and PP cases which are listed as ‘-ve’ or ‘+ve’ based on the presence of absence of T cells as determined by ELISpot analysis. [file 12879_2023_8829_MOESM1_ESM.pdf]
